# A SCOPING REVIEW OF HOME AND COMMUNITY-BASED SERVICES AND OLDER ADULTS’ HEALTH OUTCOMES

**DOI:** 10.1093/geroni/igae098.3206

**Published:** 2024-12-31

**Authors:** Seon Kim

**Affiliations:** Drexel University, Philadelphia, Pennsylvania, United States

## Abstract

**Background and Objectives:**

Most older adults aspire to age in their homes and communities rather than in institutions. However, as functional limitations grow with aging, older adults often find independent living challenging. The demand for home- and community-based services (HCBS) has steadily increased to alleviate the challenges faced by older adults. This scoping review explores the relationship between HCBS and the health outcomes of older adults.

**Methods:**

A search of the literature was conducted across major electronic databases between January 1, 2005, and August 1, 2023. 11,317 relevant peer-reviewed articles were identified, and 12 articles were selected for the final sample.

**Results:**

HCBS is consistently found to be related to better health outcomes, including physical, mental, and hospitalization. However, black and Asian older adults reported lower accessibility to HCBS and SRH compared to their White counterparts. Also, the low density and lack of HCBS in the neighborhoods are also related to the lower health of older adults.

**Discussion and Implications:**

HCBS is helpful in enhancing older adults’ health outcomes; however, the better accessibility of HCBS in neighborhoods and older individual demographic characteristics should be considered. Also, policymakers should consider addressing the diverse needs of older adults and tailor HCBS.

